# Supplementary material for: Are Hair Cortisol Levels of Humans, Cats, and Dogs from the Same Household Correlated?
Source: Animals (Basel). 2022 Jun 6;12(11):1472. doi: 10.3390/ani12111472 (PMC9179579; doi:10.3390/ani12111472)
Supplement: Supplementary file 1 [file animals-12-01472-s001.zip › animals-1740713-supplementary.pdf]

DOG

	Dog-owner interactions	at least once a day	once every few days	once a week	once a month	never
1.	How often do you kiss your dog?					
2.	How often do you play with your dog?					
3.	How often do you give your dog treats?					
4.	How often do you groom your dog?					
5.	How often do you hug your dog?					
6.	How often do you have your dog with you when you are relaxing, such as watching TV?					
	**Caregiver's emotional relationship with their dog**	definitely yes	yes	neither yes nor no	no	definitely no
7.	my dog helps me get through difficult times					
8.	my dog is beside me when I need consolation					
9.	even if everyone abandons me, my dog will always be with me					
10.	I would like to have my dog around all the time					
11.	my dog keeps me company all the time					
12.	sometimes I tell my dog things I wouldn't tell anyone else					
13.	my dog watches me all the time and focuses his attention on me					
14.	I think that the death of my dog will be a traumatic event for me					
15.	my dog gives me a reason to wake up every morning					
16.	I hope that me and my dog will never be separated					

CAT

	**Cat-owner interactions**	at least once a day	once every few days	once a week	once a month	never
1.	How often do you kiss your cat?					
2.	How often do you play with your cat?					
3.	How often do you give your cat treats?					
4.	How often do you groom your cat?					
5.	How often do you hug your cat?					
6.	How often do you have your cat with you when you are relaxing, such as watching TV?					
	**Związek emocjonalny opiekuna z kotem**	definitely yes	yes	neither yes nor no	no	definitely no
7.	my cat helps me get through difficult times					
8.	my cat is beside me when I need consolation					
9.	even if everyone abandons me, my cat will always be with me					
10.	I would like to have my cat around all the time					
11.	my cat keeps me company all the time					
12.	sometimes I tell my cat things I wouldn't tell anyone else					
13.	my cat watches me all the time and focuses his attention on me					
14.	I think that the death of my cat will be a traumatic event for me					
15.	my cat gives me a reason to wake up every morning					
16.	I hope that me and my cat will never be separated					

How often do you kiss your dog?	N	Spearman R	t(N-2)	p-value
never	11	0,035	0,104	0,919
once every few days	7	0,218	0,500	0,638
at least once a day	27	-0,008	-0,041	0,967

How often do you kiss your cat?	N	Spearman R	t(N-2)	p-value
never	13	0,686	3,128	0,0096**
once a month	4	-0,316	-0,471	0,684
once a week	5	-0,158	-0,277	0,800
once every few days	8	-0,422	-1,139	0,298
at least once a day	25	-0,206	-1,007	0,324

*** p<0,01*

How often do you play with your dog?	N	Spearman R	t(N-2)	p-value
once every few days	17	-0,014	-0,053	0,959
at least once a day	26	0,186	0,928	0,363

How often do you play with your cat?	N	Spearman R	t(N-2)	p-value
never	3	0,500	0,577	0,667
once a month	6	0,414	0,910	0,414
once a week	3	0,000	0,000	1,000
once every few days	20	-0,174	-0,748	0,464
at least once a day	23	-0,020	-0,093	0,927

How often do you give your dog treats?	N	Spearman R	t(N-2)	p-value
once every few days	17	-0,023	-0,091	0,929

at least<br>once a day	25	0,316	1,599	0,123

How often do you give your cat treats?	N	Spearman R	t(N-2)	p-value
never	3	0,500	0,577	0,667
once a month	5	0,051	0,089	0,935
once a week	14	-0,016	-0,054	0,958
once every few days	22	-0,143	-0,644	0,527
at least<br>once a day	12	0,181	0,583	0,573

How often do you groom your dog?	N	Spearman R	t(N-2)	p-value
once a month	12	0,078	0,247	0,810
once a week	9	0,836	4,024	0,005**
once every few days	22	0,078	0,348	0,731

*** p<0,01*

How often do you groom your cat?	N	Spearman R	t(N-2)	p-value
once a month	27	-0,112	-0,562	0,579
once a week	10	-0,128	-0,366	0,724
once every few days	15	-0,142	-0,519	0,612

How often do you hug your dog?	N	Spearman R	t(N-2)	p-value
once every few days	3	-0,866	-1,732	0,333
at least<br>once a day	42	0,068	0,429	0,670

How often do you hug your cat?	N	Spearman R	t(N-2)	p-value
once every few days	9	-0,439	-1,292	0,237
at least<br>once a day	43	-0,156	-1,011	0,318

How often do you have your dog with you when you are relaxing, such as watching TV?	N	Spearman R	t(N-2)	p-value
once every few days	6	0,034	0,068	0,949
at least once a day	36	0,100	0,585	0,562

How often do you have your cat with you when you are relaxing, such as watching TV?	N	Spearman R	t(N-2)	p-value
once a week	3	-0,500	-0,577	0,667
once every few days	13	0,149	0,500	0,627
at least once a day	34	-0,254	-1,483	0,148

my dog helps me get through difficult times	N	Spearman R	t(N-2)	p-value
yes	14	-0,092	-0,318	0,756
definitely yes	28	0,237	1,242	0,225

my cat helps me get through difficult times	N	Spearman R	t(N-2)	p-value
neither yes nor no	12	0,243	0,792	0,447
yes	15	0,047	0,171	0,867
definitely yes	26	-0,335	-1,740	0,095

my dog is beside me when I need consolation	N	Spearman R	t(N-2)	p-value
yes	13	-0,045	-0,151	0,883
definitely yes	29	0,321	1,758	0,090

my cat is beside me when I need consolation	N	Spearman R	t(N-2)	p-value
no	4	-0,400	-0,617	0,600
neither yes nor no	17	0,219	0,871	0,397
yes	12	-0,472	-1,694	0,121
definitely yes	22	-0,262	-1,214	0,239

even if everyone abandons me, my dog will always be with me	N	Spearman R	t(N-2)	p-value
neither yes nor no	7	0,218	0,500	0,638
yes	9	-0,350	-0,988	0,356
definitely yes	29	0,203	1,078	0,291

even if everyone abandons me, my cat will always be with me	N	Spearman R	t(N-2)	p-value
no	4	-0,400	-0,617	0,600
neither yes nor no	14	0,576	2,438	0,031*
yes	19	-0,030	-0,124	0,903
definitely yes	17	-0,347	-1,43	0,172

** p<0,05*

I would like to have my dog around all the time	N	Spearman R	t(N-2)	p-value
no	4	-0,775	-1,73	0,225
neither yes nor no	18	0,211	0,865	0,400
yes	7	0,643	1,877	0,119
definitely yes	16	0,336	1,333	0,204

I would like to have my cat around all the time	N	Spearman R	t(N-2)	p-value
no	4	-0,894	-2,828	0,106
neither yes nor no	30	0,032	0,167	0,869
yes	6	-0,754	-2,294	0,084
definitely yes	15	-0,025	-0,091	0,929

my dog keeps me company all the time	N	Spearman R	t(N-2)	p-value
no	6	-0,772	-2,43	0,072
neither yes nor no	10	0,330	0,990	0,351
yes	10	0,000	0,000	1,000
definitely yes	18	0,401	1,752	0,099

my cat keeps me company all the time	N	Spearman R	t(N-2)	p-value
no	19	-0,086	-0,354	0,727
neither yes nor no	7	0,818	3,184	0,024*
yes	13	-0,168	-0,566	0,583
definitely yes	15	-0,450	-1,818	0,092

** p<0,05*

sometimes I tell my dog things I wouldn't tell anyone else	N	Spearman R	t(N-2)	p-value
definitely no	3	0,000	0,000	1,000
no	7	-0,356	-0,851	0,434
neither yes nor no	9	0,539	1,691	0,135
yes	10	0,335	1,004	0,345
definitely yes	16	0,526	2,317	0,036*

** p<0,05*

sometimes I tell my cat things I wouldn't tell anyone else	N	Spearman R	t(N-2)	p-value
definitely no	6	-0,463	-1,044	0,355
no	13	-0,294	-1,019	0,330
neither yes nor no	8	0,037	0,091	0,931

yes	13	-0,346	-1,225	0,246
definitely yes	15	0,232	0,859	0,406

** p<0,05*

my dog watches me all the time and focuses his attention on me	N	Spearman R	t(N-2)	p-value
no	8	-0,741	-2,700	0,036*
neither yes nor no	6	0,412	0,904	0,417
yes	16	0,209	0,800	0,437
definitely yes	15	0,260	0,971	0,349

** p<0,05*

my cat watches me all the time and focuses his attention on me	N	Spearman R	t(N-2)	p-value
definitely no	3	-0,500	-0,577	0,667
no	19	0,085	0,351	0,730
neither yes nor no	11	0,285	0,892	0,395
yes	14	-0,289	-1,05	0,317
definitely yes	8	-0,539	-1,567	0,168

I think that the death of my dog will be a traumatic event for me	N	Spearman R	t(N-2)	p-value
yes	9	-0,079	-0,210	0,839
definitely yes	36	0,060	0,350	0,728

I think that the death of my cat will be a traumatic event for me	N	Spearman R	t(N-2)	p-value
yes	20	0,197	0,853	0,405
definitely yes	31	-0,303	-1,714	0,097

my dog gives me a reason to wake up every morning	N	Spearman R	t(N-2)	p-value
neither yes nor no	15	-0,009	-0,033	0,974
yes	8	0,143	0,354	0,736
definitely yes	19	0,583	2,962	0,009**

*** p<0,01*

my cat gives me a reason to wake up every morning	N	Spearman R	t(N-2)	p-value
definitely no	3	-0,866	-1,732	0,333
neither yes nor no	21	0,224	1,003	0,329
yes	13	-0,704	-3,289	0,007**
definitely yes	16	0,150	0,568	0,579

*** p<0,01*

I hope that me and my dog will never be separated	N	Spearman R	t(N-2)	p-value
neither yes nor no	4	-0,258	-0,378	0,742
yes	11	-0,023	-0,069	0,947

definitely yes	30	-0,006	-0,032	0,975

I hope that me and my cat will never be separated	N	Spearman R	t(N-2)	p-value
neither yes nor no	13	0,064	0,213	0,836
yes	16	-0,018	-0,067	0,948
definitely yes	26	-0,081	-0,397	0,695

Pair of Variables	N	Spearman R	t(N-2)	p-value
owner corisol (ng/ml) & female dog cortisol (ng/ml)	27	-0,029	-0,143	0,888
owner cortisol (ng/ml) & male dog cortisol (ng/ml)	18	0,022	0,087	0,932
owner cortisol (ng/ml) & female cat cortisol (ng/ml)	31	0,191	1,045	0,304
owner cortisol (ng/ml) & male cat cortisol (ng/ml)	24	-0,461	-2,44	0,023*
female cat cortisol (ng/ml) & female dog cortisol (ng/ml)	51	0,075	0,528	0,600
female cat cortisol (ng/ml) & male dog cortisol (ng/ml)	28	0,183	0,950	0,351
male cat cortisol (ng/ml) & female dog cortisol (ng/ml)	22	0,072	0,325	0,749
male cat cortisol (ng/ml) & male dog cortisol (ng/ml)	27	0,201	1,025	0,315

* *p<0,05*
